# Epidemiology of respiratory syncytial virus in hospitalized children over a 9-year period and preventive strategy impact

**DOI:** 10.3389/fphar.2024.1381107

**Published:** 2024-05-22

**Authors:** Lorenzo Lodi, Francesco Catamerò, Marta Voarino, Federica Barbati, Maria Moriondo, Francesco Nieddu, Walter Maria Sarli, Francesco Citera, Valeria Astorino, Caterina Pelosi, Francesca Quaranta, Silvia Stocco, Clementina Canessa, Vieri Lastrucci, Silvia Ricci, Giuseppe Indolfi, Chiara Azzari

**Affiliations:** ^1^ Department of Health Sciences, University of Florence, Florence, Italy; ^2^ Immunology Unit, Department of Pediatrics, Meyer Children’s Hospital IRCCS, Florence, Italy; ^3^ Pediatrics and Neonatology Unit, Santo Stefano Hospital, AUSL Toscana Centro, Prato, Italy; ^4^ Laboratory of Immunology and Molecular Microbiology, Meyer Children’s Hospital IRCCS, Florence, Italy; ^5^ Epidemiology Unit, Meyer Children’s Hospital IRCCS, Florence, Italy; ^6^ Department of Neurofarba, University of Florence, Florence, Italy; ^7^ Liver Unit, Department of Pediatrics, Meyer Children’s Hospital IRCCS, Florence, Italy

**Keywords:** respiratory syncytial virus, RSV, nirsevimab, prevention, children, bronchiolitis, SARS-CoV-2

## Abstract

**Background:** Respiratory Syncytial Virus (RSV) is the primary cause of respiratory infections and hospitalizations in young children globally, leading to substantial disease burden and mortality. The aim of the present study was to review and provide updates on how the SARS-CoV-2 pandemic have significantly influenced RSV epidemiology on hospitalized children due to RSV infection. A potential impact of the available preventive strategies on the same population were provided.

**Methods:** All children aged 0–6 years hospitalized at Meyer Children’s Hospital IRCCS for RSV infection from September 2014 to March 2023 were retrospectively recorded. Seasonal trends before and after SARS-CoV-2 pandemic, age distribution, ICU admission and co-infections, comorbidities and prematurity were retrieved. Predictions on the number of hospitalizations avoided by the deployment of different preventive strategies were provided.

**Results:** A total of 1,262 children with RSV infection were included in the study. The 70% of them had less than 1 year-of-age at the moment of hospitalization and almost 50% less than 3 months. In the post-pandemic seasons, a 317% increase in the number of hospitalizations was recorded with a significant increase in older children compared to the pre-pandemic seasons. ICU support was required for 22% of children, the majority of whom were under 3 months of age. Almost 16% of hospitalized children were born preterm and only 27% of hospitalized children had prior comorbidities. The rate of comorbidities among RSV hospitalized children increased with age. Nirsevimab prophylaxis could have prevented more than 46% of hospitalizations in this cohort. A preventive strategy addressing also children aged 7 months to 6 years of age with co-existing comorbidities would increase that rate above 57%.

**Discussion:** The identification of RSV hospitalization-related features is informing the decision-maker for the deployment of the wisest preventive approach on a population scale.

## 1 Introduction

Respiratory Syncytial Virus (RSV) is the leading cause of respiratory tract infections in young children globally, resulting in significant disease burden and mortality (Shi et al., 2017; Reeves et al., 2021). RSV infection represents the most frequent cause of hospitalization in children under 2 years of age, causing lower respiratory tract infections (LRTIs) such as pneumonia and bronchiolitis. Moreover, it has been associated with an increased risk of developing recurrent wheezing and asthma (Zurita-Cruz et al., 2020; Henderson et al., 2005). Currently, there is no specific treatment for RSV infection.

Following the SARS-CoV-2 pandemic, it has been observed a surge in annual RSV-related LRTIs and consensual hospital admissions, attributed to the rise in immunologically naïve patients after the lockdown years (Rice et al., 2023; Viñeta Paramo et al., 2023). The resurgence in RSV-associated hospital admissions urged the scientific community and policymakers to implement preventive strategies, aiming to alleviate the disease burden on healthcare systems and the non-financial burden on patients and their families. Until 2022, the only preventive strategy available for children was Palivizumab (Synagis™ - AstraZeneca), a neutralizing-RSV humanized IgG1 monoclonal antibody. However, due to its short half-life, Palivizumab requires monthly injections, for a total of 5 administrations (Robbie et al., 2012) to maintain passive immunization all along the epidemic season. Indeed, partial seasonal dosing was associated with a higher risk of RSV-related hospitalization (La Via et al., 2013). Moreover, due to its high cost, this prophylactic strategy was reserved for high-risk children (e.g., premature infants and children with severe pulmonary or cardiac comorbidities) so that it ultimately did not affect the epidemiological trends of the infection as most RSV-related hospitalization occurred in otherwise healthy infants (Azzari et al., 2021; Barbati et al., 2020).

As of today, several other monoclonal antibodies are under development, but the only one licensed is Nirsevimab (Beyfortus™, AstraZeneca/Sanofi Pasteur), a long-acting humanized monoclonal antibody targeting RSV’s fusion protein that has been recently approved for passive immunization of infants during their first RSV season by the United States Food and Drugs Administration (FDA) and the European Medicines Agency (EMA). This approval was based on the results of three randomized controlled trials demonstrating its efficacy and safety in preventing severe RSV infection in both healthy and preterm children after a single dose per epidemic season (MELODY phase III, MEDLEY phase II and a phase IIb trial) (Griffin et al., 2020; Domachowske et al., 2022; Hammitt et al., 2022). The United States and some European countries already implemented universal immunization of children during their first RSV season and of high-risk children <18 or <24 months old. Preliminary published data from the immunization campaign in the Spanish region of Galicia revealed an excellent population coverage and effectiveness (Martinón-Torres et al., 2023).

FDA and EMA have approved an adjuvanted recombinant vaccine (Arexvy™, GlaxoSmithKline) for the active immunization of the elderlies, and a non-adjuvanted recombinant vaccine (Abrysvo™, Pfizer) for both elderlies and pregnant women. However, currently there are no vaccines available for the active immunization of pediatric population (Topalidou et al., 2023).

The aim of our study was to describe RSV epidemiology in a pediatric population of children hospitalized due to RSV infection before and after SARS-CoV-2 pandemic. We aimed to identify those pediatric patients at higher risk of hospitalization and severe course to inform the decision-maker and deploy the wisest RSV preventive strategy in the real-life setting of Tuscany region, Italy.

## 2 Materials and methods

### 2.1 Study design

This observational study retrospectively evaluated all children between 0 and 6 years of age included in the Molecular Surveillance Register who had been admitted to the Meyer’s Children Hospital IRCSS (Florence, Italy) with a diagnosis of RSV infection from September 2014 to March 2023.

The study analyzed and compared the seasonal trends of RSV infection, its distribution according to age and the clinical and laboratory data of children hospitalized due to RSV infection over a 9-years period divided in two different time frames: the period before the SARS-CoV-2 pandemic (pre-pandemic period: September 2014-March 2020) and the period after the beginning of the pandemic (post-pandemic period: April 2020-March 2023).

### 2.2 Inclusion and exclusion criteria and data collection

Patients under 6 years of age admitted to the Meyer’s Children Hospital IRCCS between September 2014 and March 2023 with a clinical diagnosis of respiratory symptoms and a positive test result for RSV were eligible for inclusion. All patients hospitalized for issues other than RSV-related respiratory distress (e.g., Oncological, Orthopedic, Surgical, etc.) with a positive swab for RSV were excluded from the study.

Clinical and laboratory data were recorded from the electronic medical charts of patients using an anonymized standard report form. Clinical data included gender assigned at birth, age at admission, comorbidities, gestational age, duration, setting of hospitalization and outcome. Gestational age was divided according to Istituto Superiore di Sanità (ISS) as it follows: preterm <37 weeks of gestational age; late preterm between 36 + 6 and 34 weeks of gestational age; moderate preterm between 33 + 6 and 32 weeks of gestational age; very preterm between 31+6 weeks of gestational age; extremely preterm <28 weeks of gestational age.

Comorbidities were divided into the following categories: recurrent wheezing, congenital heart diseases, bronchopulmonary dysplasia (BPD), neurological impairment, immune abnormalities, oncological diseases, cystic fibrosis, trisomy 21 and genetic disorders (including all the chromosomic abnormalities beyond trisomy 21 and diseases associated to specific point mutations). Patients with comorbidities not fitting the previously mentioned categories were classified as presenting “other comorbidities” (congenital malformations, endocrinological abnormalities, urinary tract anomalies, laryngomalacia, congenital infections, gastrointestinal disorders, hematological disorders or febrile seizures).

Laboratory data included any documented co-infections with respiratory tract pathogens other than RSV.

### 2.3 Laboratory methods

RSV positivity was assesses using Polymerase Chain Reaction (PCR) on nasopharyngeal swab or on bronchoalveolar lavage. The test for RSV was requested alone or within an extended panel of Influenza-like-Illness (ILI) which included Influenza virus, Parainfluenza virus, Bocavirus, Rhinovirus, Adenovirus and Metapneumovirus. Since epidemic season 2020/2021 SARS-CoV-2 was routinely tested in each patient before admission.

Data from seasons 2014–2019 included only the positivity of the test for RSV since, at that time, the extended panel for the research of the previously highlighted viruses via PCR on nasal swabs was not yet available.

Details on the method used for the detection of RSV together with the other viruses of the ILI panel and for the detection of SARS-CoV-2 are reported in the Supplementary Material.

### 2.4 Statistical analysis

Statistical analyses were performed using SPSS (version 29.0.1.0, IBM Corp, Endicott, New York) Data were reported as mean and standard deviation (SD) and as median and interquartile ranges (IQR), for continuous variables, and as number (percentage, %) for categorical variables. We compared the categorical variables using the Chi-squared or Fisher’s exact test, depending on the number of observations. Two-sided *p*-values <0.05 were considered statistically significant.

### 2.5 Ethics

All data included in this study were obtained as part of routine clinical activity and evaluated retrospectively and anonymously in the study. The study was approved by the Pediatric Ethical Committee on the 2^nd^ of November 2021 (VRS_PED, number 285/2021).

## 3 Results

### 3.1 Population, seasonal trend and age distribution

Data from 1,262 children (691 male-assigned-at-birth, 54.8%) under 6 years of age (median age 4.0 months, IQR: 1.0–15.2 months) hospitalized for RSV infection at Meyer Children’s Hospital IRCSS from November 2014 to March 2023 were retrospectively retrieved. Overall, about 70% of cases (874/1262, 69.2%) were under 1 year of age at the moment of hospitalization with the majority of them being under 3 months of age (611/874, 69.9%;Supplementary Figure S1 of Supplementary Material). Age-related case distribution per epidemic season is detailed in Table 1. The number of cases occurred in patients older than 1 year was significantly higher in the post-pandemic seasons (255/645, 39.5%) when compared to the pre-pandemic seasons (131/610, 21.5%; *p* < 0.0001). Similarly, within the first year of life, the number of patients older than 3 months of age was significantly higher in the post-pandemic seasons (191/390, 49.0%) compared to the pre-pandemic ones (166/479, 34.7%; *p* < 0.0001).

**TABLE 1:** Distribution of registered cases according to age (in years) and epidemic season, described as an absolute number and percentage over the total cases [n] recorded in the given season. The “POST COVID-19” column shows the total cases of season 2021–2022 to 2022–2023 and the “PRE-COVID-19” those of season 2014–2015 to season 2019–2020.

Age (years)	Post COVID-19 (n/%) [n = 645]	20/21 (n/%) [n = 7]	PRE COVID-19 (n/%) [n = 610]
0–1	390 (60.4%)	5 (71.4%)	479 (78.5%)
1–2	106 (16.4%)	1 (14.2%)	78 (12.8%)
2–3	65 (10.1%)	1 (14.2%)	26 (4.3%)
3–4	48 (7.4%)	0 (0%)	14 (2.3%)
4–5	24 (3.7%)	0 (0%)	7 (1.1%)
5–6	12 (1.9%)	0 (0%)	6 (1%)

**FIGURE 1:**
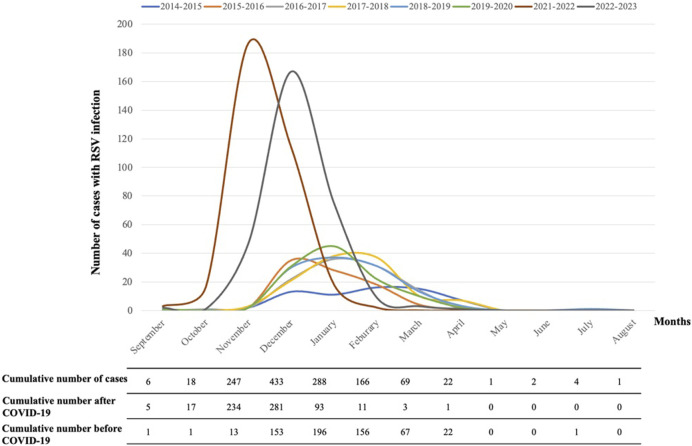
Seasonal trend of RSV infection in Tuscany in each epidemic season and cumulative number of cases, before and after the SARS-CoV-2 pandemic.

Almost no RSV cases were recorded during the SARS-CoV-2 pandemic (2020/2021). In post-pandemic, the number of RSV-related hospitalizations showed a 317% increase per season (645 cases in two seasons) compared to pre-pandemic (610 cases in six seasons). RSV circulation in seasons 2021–2022 and 2022–2023 began earlier (October), presented an earlier peak (December) and ended earlier (February) compared to previous seasons (Figure 1). In post-pandemic years, 515/645 cases (79.8%) were recorded between November and December, whereas the peak in the pre-pandemic years (505/610 cases, 82.7%) was recorded between December and February. Interestingly, the 7 cases of RSV infection recorded during the year of lockdown (2020–2021) were in the summertime. The details of the seasonal trend of RSV infection are shown in Figure 1.

### 3.2 Intensive care unit (ICU) and co-infections

ICU support was necessary for 282/1262 (22.3%) hospitalized children. When considering children hospitalized in the post pandemic period the proportion of hospitalized patients requiring ICU support reaches a striking 25.3% (163/645). The great majority of ICU-hospitalized children were below 1 year of age at the moment of hospitalization (224/282, 79.4%), with 168/282 (59.6%) under 3 months of age and 87/282 (30.8%) under 1 month. One patient (1/1262, 0.08%) over the entire study period died during RSV infection.

The presence of a viral co-infection in the respiratory tract was sought in those cases recorded between winter 2019 and spring 2023 (Supplementary Table S1 of Supplementary Material). The total number of patients presenting a viral co-infection was 278/769 (36.1%). The most frequent viral co-infections were caused by Adenovirus, Rhinovirus and Influenza virus. There was no statistically significant difference between ICU-admitted patients presenting with RSV infection alone 111/189 (58.7%) and those with concomitant respiratory viral co-infection 78/189 (41.3%) that were even less represented.

### 3.3 Comorbidities and prematurity

Almost 16% (201/1262) of hospitalized children were born before 37 weeks w) of gestational age but only 51.2% (103/201) were in their first RSV epidemic season. The mean age of preterm patients (<37w) at the time of the hospitalization was 14.3 months (IQR 2–21 months). Of these 201 preterm children, 107 were late preterm (53.2%), 35 (17.4%) were moderate preterm, 33 (16.4%) were very preterm and 26 (12.9%) were extremely preterm. The details of the distribution of the cases according to gestational age in different seasons are shown in Supplementary Table S2 of Supplementary Material.

Due to the heterogeneity of Palivizumab indications in different countries a gestational age lower than 32w was defined as a cut-off for its administration in our setting determining a reduction in the number of RSV hospitalization in premature infants born before 32w. In Italy, according to ISS the ratio between preterm children born between 32w and 36w+6 and those born before 32 is 5.9, while, for the above mentioned reason, in our cohort of hospitalized preterm at their first RSV season the ratio is higher (7.6).

One or more comorbidities beyond prematurity known to possibly exacerbate RSV-infection course were detected in 342/1262 (27.1%) patients, with 34/342 (9.9%) having multiple comorbidities: 30/342 (8.8%) two comorbidities and 4/342 (1.1%) three or more comorbidities. In particular, 92 patients (7.3%) had recurrent wheezing, 70 patients (5.5%) congenital heart diseases, 43 (3.4%) neurological impairment, 39 (3.1%) genetic disorders, 18 (1.4%) bronchopulmonary dysplasia, 10 (0.8%) trisomy 21, 8 (0.6%) oncological disease, 6 (0.5%) immune abnormalities, 5 (0.4%) cystic fibrosis and 51 (4%) other comorbidities (Supplementary Figure S2 of Supplementary Material; Supplementary Table S3 of Supplementary Material).

**FIGURE 2:**
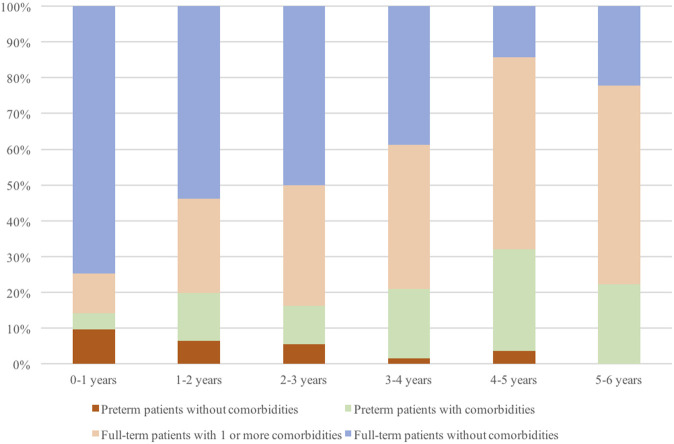
Percentage of full-term or preterm patients with or without comorbidities according to age (in years), over all patients included in the study.

The rate of comorbidities including prematurity increased with the age of the patients being slightly above 25% in patients with less than 1 year of age, reaching 50% between 2 and 3 years and above 85% in children between 4 and 5 years of age. Among these 85% of patients, about 30% were children born preterm. Preterm patients presenting comorbidities were more represented in every age group compared to those preterm patients without other comorbidities (Figure 2).

In total, 198/1262 (15.7%) children between 7 months and 6 years old presented one or more comorbidities excluding those categorized as others.

### 3.4 RSV immunization strategies impact

**FIGURE 3:**
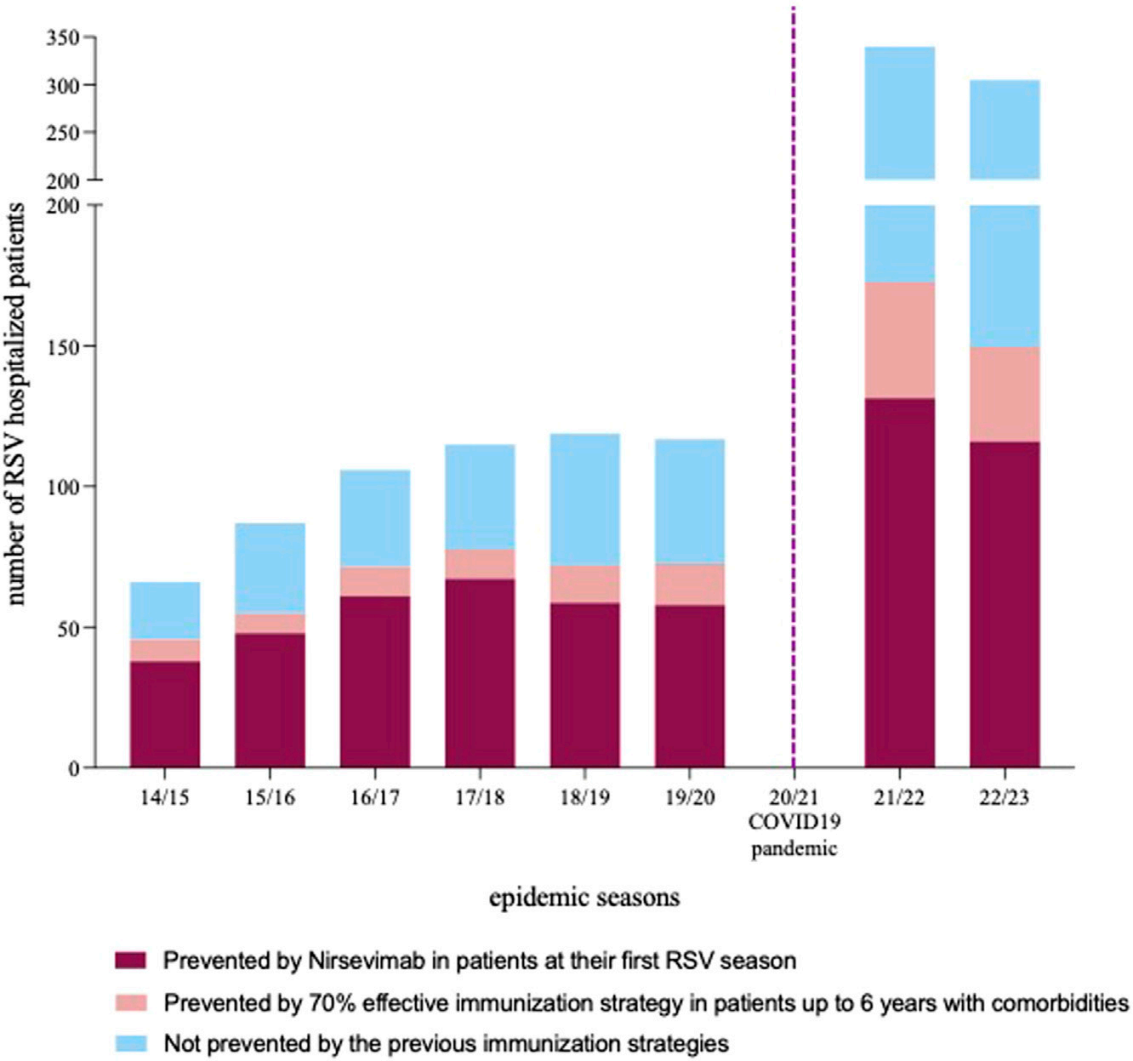
Number of RSV hospitalization per epidemic season. Columns are divided in segments representing the number of hospitalization potentially prevented by nirsevimab in patients at their first RSV season (bottom segment), by an additional immunization strategy with a postulated efficacy of 70% targeting patients with comorbidity up to 6 years of age (intermediate segment) and hospitalizations that would not have been preventive by the two previous preventive strategies.

Based on the Italian eligibility criteria for Nirsevimab, 750/1262 (59.4%) children in our cohort would have benefit from passive prophylaxis being neonates and infants in their first RSV season (0–6 months). Considering a target population coverage of virtually 100% and the estimated Nirsevimab efficacy in preventing hospitalization of approximately 77.3% (CI 50.3%–89.7%) (Simões et al., 2023), 580/1262 (45.9%) patients (CI 377–672 children, corresponding to 29.8% and 53.2% respectively) could have avoided hospitalization (details in Figure 3). Given that 202 out of 750 children under 6 months of age required ICU admission, Nirsevimab would have prevented 156 (CI 101–181) ICU admissions, corresponding to a remarkable 55% (156/282) of the total ICU admissions in our study period.

When including children aged up to 24 months presenting comorbidities known to expose to a more severe course of RSV disease according to Galician eligibility criteria for expanded access (44 patients, with 27% of them requiring ICU support), the estimate of hospitalizations prevented would raise to 48.7% (614/1262) (CI 399–672 children, corresponding to 31.6% and 53.2% respectively).

Moreover, when examining alternative preventive approaches (with an estimated efficacy of 70%), the inclusion of patients between 7 months and 6 years of age, and presenting at least one comorbidity, could have potentially averted 139 (11%) hospitalizations.

## 4 Discussion

After the SARS-CoV-2 pandemic, a shift in the seasonal pattern of RSV infections was observed. This change was marked by a significant increase in the annual number of RSV-related LRTIs and by the involvement of older children. These phenomena are presumably linked to the increase of immunologically naïve children susceptible to RSV infection, generated by the reduced circulation of the virus as a consequence of the social health interventions applied for the containment of SARS-CoV-2 epidemic (Rice et al., 2023). Additionally, in our setting, the rise in the median age of affected patients is influenced by the introduction since 2019 in clinical routine practice of an extended PCR panel for the diagnosis of influenza-like illnesses for all patients presenting with respiratory symptoms. This allowed to detect the infection in older children that previously were not routinely investigated for RSV and underscores the importance of promoting extended panel search for ILI in order to understand the epidemiological trends of different viral respiratory infections that would otherwise be clinically indistinguishable. Our study reveals that the primary burden of the infection still affects children under 1 year of age, with infants in their first 3 months being the most susceptible. However, the circulation of the virus among cohorts of older children may negatively impact the containment of the infection at a population level, emphasizing the need for more comprehensive preventive strategies.

In the existing literature, there is considerable variability among different studies in the percentage of patients requiring ICU support during RSV infection, ranging from 3% in previous studies (Resch et al., 2002) to 16% in more recent ones (Resch et al., 2020). In our setting, more than 20% of hospitalized patients required ICU support, with the vast majority being under 1 year of age, particular clustering below 3 months of age, confirming that young age is a major susceptibility factor for a more severe course of the infection. Moreover, our study corroborated the findings of the study conducted by Rao et al. in Colorado (Rao et al., 2023), U.S., revealing an increase in patients requiring ICU admission in the post-pandemic period, with a striking 25.3% of ICU admission. This rate could eventually be influenced by the significant surge in RSV cases in the last seasons and on the consequent pressure exerted on healthcare facilities. The mortality of RSV infection was 0.08% in accordance to previous studies conducted in high-resource settings (Bont et al., 2016). Interestingly, the presence of viral co-infection compared to RSV infection alone was not associated with a more severe course of the disease and ICU admission, in accordance with previous studies (Li et al., 2020).

The majority of hospitalized patients were otherwise healthy full-term children, with young age being the most important susceptibility factor for RSV-related LRTIs. The comorbidity more represented among hospitalized patients was prematurity, with 16% of them born preterm, and more than half of them being late preterm. It is important to highlight that the median age of hospitalized premature infants was considerably higher than the median age of the entire patient cohort, as prematurity represents a susceptibility factor for the disease extending beyond their first RSV season. The other main comorbidities presented among hospitalized patients were recurrent wheezing, congenital heart diseases and neurological impairment. Among older children, as the susceptibility factor of young age fades, there is an increasing rate of comorbidities contributing to the susceptibility to the infection. Indeed, in our cohort, more than 85% of patients older than 4 years of age presented comorbidities, with prematurity still being the most represented among them.

The Italian drug administration agency AIFA (Agenzia Italiana del Farmaco) approved Nirsevimab for the prevention of RSV lower respiratory tract disease in neonates and infants during their first RSV season. The implementation of Nirsevimab with the current eligibility criteria would have prevented 46% of hospitalizations in our cohort. Even with the extension of eligibility criteria to patients between 7 and 24 months of age with comorbidities, as outlined by the Galician immunization program, there would be only a minimal increase in the percentage of prevented hospitalizations. However, it is crucial to note that up to 27% of patients in this category required ICU admission. Moreover, these figures are likely underestimated as the reduced circulation of the virus would further diminish the rate of infection at a population level and subsequently the rate of hospitalized children. Considering the remarkably increased representation of patients with comorbidities among hospitalized older children, a preventive strategy targeting children from 7 months to 6 years of age with at least one comorbidity could have prevented an addition 11% of hospitalization, assuming an efficacy of at least 70%. Moreover, an immunization campaign targeting patients with comorbidities could be integrated into their individual follow-up, eliminating the financial and non-financial costs of additional access to healthcare facilities. To target these different populations, it would be desirable to deploy combined preventive strategies including both passive and active immunization. Passive prophylaxis confers rapid protection in the first months of life but cannot provide long-lasting protection against RSV infection beyond a single epidemic season. Consequently, children who received Nirsevimab during their initial RSV season will remain immunologically naïve in their second one. Hence, it becomes crucial to closely monitor epidemiological trends following the introduction of population-wide passive immunization in the first year of life and, based on eventual changes of the RSV burden in older immunologically naïve children, to ultimately implement active immunization strategies for children entering their second RSV season. Currently, the use of vaccines against RSV in the pediatric population is still under study (e.g., NCT03387137, NCT03227029). Theoretically, the combination of passive immunization, administering Nirsevimab to all children at their first RSV season, and active immunization even with a vaccination campaign directed to patients up to 6 years of age with at least one comorbidity could prevent almost 60% of severe RSV infection requiring hospitalization in our setting. Wider population targets could eventually produce and additional impact on the circulation of the virus on a population scale, contributing to a more significant reduction of the RSV burden.

## Data Availability

The raw data supporting the conclusion of this article will be made available by the authors, without undue reservation.
