# Supplementary material for: Epidemiology of respiratory syncytial virus in hospitalized children over a 9-year period and preventive strategy impact
Source: Front Pharmacol. 2024 May 22;15:1381107. doi: 10.3389/fphar.2024.1381107 (PMC11150665; doi:10.3389/fphar.2024.1381107)
Supplement: Supplementary file 1 [file DataSheet1.docx]

**Table S1**

|  | TOTAL (n/%) [n = 769] | 22/23 (n/%) [n = 305] | 21/22 (n/%) [n = 340] | 20/21 (n/%) [n = 7] | 19/20 (n/%) [n = 117] |
| --- | --- | --- | --- | --- | --- |
| Total Co-infection detected | 278 (36.1%) | 100 (32.8%) | 151 (44.4%) | 0 (0%) | 27 (23.1%) |
| Total ICU patients | 189 (24.6%) | 77 (25.2%) | 86 (25.3%) | 1 (14.2%) | 25 (21.3%) |
| ICU-patients with RSV alone | 111 (14.4%) | 51 (16.7%) | 43 (12.6%) | 1 (14.2%) | 16 (13.7%) |
| ICU patients with RSV + Co-infection | 78 (10.1%) | 26 (8.5%) | 43 (12.6%) | 0 (0%) | 9 (7.7%) |

**Table S1**. Distribution of total viral co-infections and cases admitted to ICU (total, with and without coinfections) according to Seasons (from 2019-2020 to 2022-2023); the column “TOTAL” sums the cases from 2019-2020 to 2022-2023 seasons. Cases are described as an absolute number and percentage over the total cases [n] registered in the given season. ICU = Intensive Care Unit.

**Table S2**

|  | <28w | 28 -31w+6d | 32 -33w+6d | 34-36w+6d | Total preterm |
| --- | --- | --- | --- | --- | --- |
| 22/23 (n/%) [n = 305] | 7  (2.3%) | 6  (2%) | 1  (0.3%) | 25  (8.2%) | 39  (12.8%) |
| 21/22 (n/%) [n = 340] | 8  (2.3%) | 10  (2.9%) | 12  (3.5%) | 24  (7%) | 54  (15.9%) |
| 20/21 (n/%) [n = 7] | 0  (0%) | 1  (14.3%) | 0  (0%) | 0  (0%) | 1  (14.3%) |
| 19/20 (n/%) [n = 117] | 2  (1.7%) | 4  (3.4%) | 5  (4.3%) | 7  (5.9%) | 18  (15.4%) |
| 18/19 (n/%) [n = 119] | 1  (0.8%) | 2  (1.6%) | 4  (3.2%) | 13  (10.9%) | 20  (16.8%) |
| 17/18 (n/%) [n = 115] | 3  (2.6%) | 0  (0%) | 3  (2.6%) | 14  (12.1%) | 20  (17.4%) |
| 16/17 (n/%) [n = 106] | 4  (3.8%) | 6  (5.7%) | 4  (3.8%) | 10  (9.4%) | 24  (22.6%) |
| 15/16 (n/%) [n = 87] | 1  (1.1%) | 2  (2.3%) | 6  (6.9%) | 9  (10.3%) | 18  (20.7%) |
| 14/15 (n/%) [n =66] | 0  (0%) | 2  (3%) | 0  (0%) | 5  (7.6%) | 7  (10.6%) |
| TOTAL(n/%)  [n =1262] | 26  (12.9%) | 33  (16.4%) | 35  (17.4%) | 107  (53.2%) | 201  (15.9%) |

**Table S2.** Distribution of registered cases according to gestational age (in weeks) and season, described as an absolute number and percentage over the total cases [n] recorded in the given season. The column “total preterm” shows all preterm patients born < 37 weeks of gestational age. w: weeks of gestational age; d: days of gestational age.

|  | TOTAL (n/%) [n = 1262] | 22/23 (n/%) [n = 305] | 21/22 (n/%) [n = 340] | 20/21 (n/%) [n = 7] | 19/20 (n/%) [n = 117] | 18/19 (n/%) [n = 119] | 17/18 (n/%) [n = 115] | 16/17 (n/%) [n = 106] | 15/16 (n/%) [n = 87] | 14/15 (n/%) [n = 66] |
| --- | --- | --- | --- | --- | --- | --- | --- | --- | --- | --- |
| Congenital heart disease | **70 (5.5%)** | 15  (4.9%) | 20 (5.9%) | 0  (0%) | 5  (4.3%) | 10 (8.4%) | 5  (4.4%) | 6  (5.7%) | 3  (3.4%) | 6  (9.2%) |
| Neurological impairment | **43 (3.4%)** | 13  (4.3%) | 14 (4.1%) | 0  (0%) | 3  (2.6%) | 4  (3.3%) | 2  (1.8%) | 3  (2.9%) | 3  (3.4%) | 1  (1.5%) |
| Immunological disease | **6 (0.5%)** | 2  (0.7%) | 1  (0.3%) | 0  (0%) | 0  (0%) | 0  (0%) | 1  (0.9%) | 2  (1.9%) | 0  (0%) | 0  (0%) |
| Oncological disorders | **8 (0.6%)** | 3  (1%) | 2  (0.6%) | 0  (0%) | 1  (0.8%) | 1  (0.7%) | 0  (0%) | 1  (0.9%) | 0  (0%) | 0  (0%) |
| Cystic fibrosis | **5 (0.4%)** | 0  (0%) | 2  (0.6%) | 0  (0%) | 0  (0%) | 0  (0%) | 1  (0.9%) | 1  (0.9%) | 1  (1.1%) | 0  (0%) |
| Wheezing | **92 (7.3%)** | 22  (7.2%) | 18 (5.3%) | 0  (0%) | 15 (12.8%) | 6  (5%) | 9  (7.8%) | 6  (5.7%) | 6  (6.8%) | 10 (15.4%) |
| trisomy 21 | **10 (0.8%)** | 3  (1%) | 3  (0.9%) | 0  (0%) | 0  (0%) | 1  (0.7%) | 1  (0.9%) | 1  (0.9%) | 1  (1.1%) | 0  (0%) |
| Genetic disorders | **39 (3.1%)** | 7  (2.3%) | 13 (3.8%) | 0  (0%) | 5  (4.3%) | 3  (2.6%) | 0  (0%) | 5  (4.8%) | 2  (2.3%) | 4  (6.1%) |
| Bpd | **18 (1.4%)** | 3  (1%) | 5  (1.5%) | 0  (0%) | 3  (2.6%) | 1  (0.7%) | 1  (0.9%) | 1  (0.9%) | 2  (2.3%) | 2  (3.1%) |
| Others | **51 (4%)** | 13  (4.3%) | 17  (5%) | 0  (0%) | 4  (3.4%) | 6  (5%) | 4  (3.5%) | 3  (2.9%) | 3  (3.4%) | 1  (1.5%) |

**Table S3**

**Table S3.** Distribution of the comorbidities according to different seasons, described as an absolute number and percentage over the total cases [n] registered in the given season. The column “total” shows all the cases from 2014-2015 to 2022-2023 seasons. BPD = bronchopulmonary dysplasia.

**Figure S1.** Total number of patients <1 year-old included in the study, divided by age in months. m = months of age.

**Figure S2**

**Figure S2**: Distribution of the different comorbidities over the total number of patients presenting comorbidities.

**Supplementary Methods**

The analysis was performed on a nasal swab or bronchoalveolar lavage fluid. Viral RNA was extracted from biological samples using MagCore® Viral Nucleic Acid Extraction Kit (Diatech LabLine, Jesi, Italy). The quantitative Reverse Transcription Polymerase Chain Reaction (RT-qPCR) amplification was performed in 20 μl reaction volumes containing 5 μl of TaqPath 1-Step Multiplex Master Mix (Applied Biosystem, Waltham, Massachusetts, USA). The RT-qPCR was performed in an QuantStudio 5 Real-Time PCR System (Applied Biosystem, Waltham, Massachusetts, USA) using the following cycling parameters: 25°C for 2 minutes, 53 °C for 10  minutes, 95°C for 2  minutes followed by 40 cycles of a two-stage temperature profile of 95 °C for 3 seconds and 60 °C for 30 seconds.

Positive and negative controls were used to validate each run. All reactions were performed in duplicate. If no increase in fluorescent signal was observed after 40 cycles, the sample is assumed as negative for the analyzed pathogen.

*RT-qPCR panel for ILI detection*

The ILI panel included RSV, Influenza virus (A, B), Parainfluenza virus (serotype 1, 2, 3), Adenovirus, Bocavirus, Metapneumovirus and Rhinovirus. Primers and probes are listed in the Supplementary Table S4. All primers were used at a concentration of 300 nM; the FAM-labelled probe was used at a concentration of 25 nM and VIC-labelled probe was used at a concentration of 50 nM. Five μl of RNA extract was used for each reaction. The presence of SARS-CoV-2 was evaluated separately through RT-qPCR according to international guidelines (1).

|  |  | **Forward** | **Reverse** | **Probe** |
| --- | --- | --- | --- | --- |
| Mix1 | **Flu H1N1** | CAAGCCGGAAATAGCAATAAGAC | CCCGGCTCTACTAGTGTCCAGTA | VIC-CAAAGTGAGGGATCAAGAAGGGAGAATGAAC-QSY |
|  | **Flu H3N2** | GATTATGCCTCCCTTAGGTCACTAGT | TCCAGTCCAATTGAAGCTTTCA | FAM-CCTCATCCGGCACACTGGAGTTTAACA-QSY |
| Mix2 | **Flu B** | AAA ACT AGG AAC GCT CTG TGC TTT | GATCTCGCTGCTCTGCTATGAG | VIC-TGCGAGAAACAAGCATCACATTCACACA-QSY |
|  | **RSV** | GGAAACATACGTGAACAARCTTCA | CATATTGTWAGTGATGCAGGATCAT | FAM-AAGGCTCCACATACACAGCTGCTGT-QSY |
| Mix3 | **Adenovirus** | GGACGCCTCGGAGTACCTGAG | ACNGTGGGGTTTCTGAACTTGTT | VIC-CTGGTGCAGTTCGCCCGTGCCA-QSY |
|  | **Rhinovirus** | CCCGTGTGCTCRCTTTG | CTAGCTGCAGGGTTARGGTTAGC | FAM- TCCTCCGGCCCCTGAATGT-QSY |
| Mix4 | **PIV1** | ATCTCATTATTACCYGGACCAAGTCTACT | CATCCTTGAGTGATTAAGTTTGATGAATA | FAM-AGGATGTGTTAGAYTACCTTCATTATCAATTGGTGATG-QSY |
|  | **PIV2** | CTGCAGCTATGAGTAATC | TGATCGAGCATCTGGAAT | VIC-AGCMATGCATTCACCAGAAGCCAGC-QSY |
| Mix5 | **PIV3** | ACTCTATCYACTCTCAGACC | TGGGATCTCTGAGGATAC | VIC-AAGGGACCACGCGCTCCWTTCATC-QSY |
|  | **Metapneumovirus** | AACCGTGTACTAAGTGATGCACTC | CATTGTTTGACCGGCYCCATAA | FAM-CTTTGCCATACTCAATGAACAAAC-QSY |
| Mix6 | **Bocavirus** | AGAGGCTCGGGCTCATATCA | GAGGTCTTCGAAGCAGTGCAA | VIC-CAGGAACACCCAATCAGCCACCTATCG-QSY |
|  | **B. pertussis** | CGGATGAACACCCATAAGCAT | CGATCAATTGCTGGACCATTT | FAM-CCCGATTGACCTTCCTACGTCGACTC-QSY |

**Supplementary Table 1.** Primer and probe sequences for the RT-qPCR analysis of the ILI panel

RT-qPCR: Quantitative Reverse Transcription Polymerase Chain Reaction; Flu: Influenza virus; RSV: Respiratory Syncytial Virus; PIV: parainfluenza virus; B. pertussis: Bordetella pertussis

**Reference:**

1. Centers for Disease Control and Prevention. Interim Guidelines for Collecting and Handling of Clinical Specimens for COVID-19 Testing. Available from: <https://www.cdc.gov/coronavirus/2019-nCoV/lab/guidelines-clinical-specimens.html>. Accessed 17 January 2022.
